# Quantitative Analysis of 92 12-Week Sub-elite Marathon Training Plans

**DOI:** 10.1186/s40798-024-00717-5

**Published:** 2024-05-02

**Authors:** Melanie Knopp, Daniel Appelhans, Martin Schönfelder, Stephen Seiler, Henning Wackerhage

**Affiliations:** 1 Department of Health and Sport Sciences, Technical University of Munich, Connollystraße 32, 80809 Munich, Germany; 2 adidas Innovation, adidas AG, Adi-Dassler-Str. 1, 91074 Herzogenaurach, Germany; 3 Faculty of Health and Sport Sciences, University of Agder, Kristiansand, Norway

**Keywords:** Marathon, Running, Endurance, Recreational athletes, Training guidelines

## Abstract

**Background:**

A typical training plan is a mix of many training sessions with different intensities and durations to achieve a specific goal, like running a marathon in a certain time. Scientific publications provide little specific information to aid in writing a comprehensive training plan. This review aims to systematically and quantitatively analyse the last 12 weeks before a marathon as recommended in 92 sub-elite training plans.

**Methods:**

We retrieved 92 marathon training plans and linked their running training sessions to five intensity zones. Subsequently, each training plan was grouped based on the total running volume in peak week into high (> 90 km/week), middle (65–90 km/week), and low (< 65 km/week) training volume plan categories.

**Results:**

In the final 12 weeks before a race, recommended weekly running volume averaged 108 km, 59 km, and 43 km for high, middle, and low distance marathon training plans. The intensity distribution of these plans followed a pyramidal training structure with 15–67–10–5–3%, 14–63–18–2–3%, and 12–67–17–2–2% in zones 1, 2, 3, 4, and 5, for high, middle, and low volume training plans, respectively.

**Conclusions:**

By quantitatively analysing 92 recommended marathon training plans, we can specify typical recommendations for the last 12 weeks before a marathon race. Whilst this approach has obvious limitations such as no evidence for the effectiveness of the training plans investigated, it is arguably a useful strategy to narrow the gap between science and practice.

**Supplementary Information:**

The online version contains supplementary material available at 10.1186/s40798-024-00717-5.

## Key points

- This review links science and best practice recommendations by quantitatively analysing 92 publicly available marathon training plans for sub-elite marathon runners.
- Weekly planned running distance in the last 12 weeks before the marathon ranged from 107.7 km for high volume, 58.5 km for middle volume, to 42.9 km for low volume training plans, with the longest run in these plans ranging from 35.2 km for high to 30.9 km for low volume plans.
- Following a five-zone intensity model, training intensity distribution for all volume categories followed a low, middle, and high intensity pyramidal structure with 13.2% in zone 1, 65.6% in zone 2, 15.1% in zone 3, 3.0% in zone 4, and 3.0% in zone 5. Most of the training volume was recommended to be run in zone 2.

**Supplementary Information:**

The online version contains supplementary material available at 10.1186/s40798-024-00717-5.

## Background

Recreational marathon training is popular, attracting individuals of various fitness levels and backgrounds who aspire to complete the iconic 42.2 km race [1–3]. When it comes to training for a marathon, a major challenge is that the training plan is not just one intervention but a complex mix of many interventions such as runs of different intensities and distances as well as nutritional interventions such as carbo-loading, or recovery techniques [4]. This mix of training forms is then applied over months, and it changes with time due to periodisation and tapering [4]. In contrast to a single medical intervention such as a drug treatment, it is practically impossible to investigate in a well-controlled, randomised trial, whether, for example, a specific 3-month marathon training intervention is more effective than a control marathon training intervention. An analysis of the scientific knowledge available for training advice to long-distance runners and coaches reveals limitations. These include a lack of research specifically with trained distance runners and methodological challenges that make it hard to interpret the findings. As a result, the analysis cautions giving training recommendations based on the limited available scientific knowledge [5]. Due to this problem, current marathon training plans are mostly experience-based, occasionally supplemented by evidence-based recommendations such as those related to carbohydrate ingestion.

To address this “evidence problem” of training practice, several researchers have developed new concepts of generating evidence or of utilising evidence for writing training plans. For example, Wackerhage and Schoenfeld have proposed evidence-informed training plans, where some of the decisions are based e.g., on meta-analyses and systematic reviews whereas others are based on subjective best practice [4]. Another notion for elite training plans is the concept of results-proven practice presented by Haugen and colleagues. This approach involves gathering and analysing training plan data from elite athletes who have attained top-tier outcomes [6]. Whilst the lack of comparison and control in these plans means that we cannot determine whether an athlete has won a championship or Olympic medal because of or despite the training plan used, we can infer that the training plan allowed an elite athlete to attain exceptional results. To date, Haugen et al. [6–8] have published results-proven practice reviews on sprinting, middle-distance, and long-distance running that give a practically useful insight into the training strategies of elite athletes. The resultant information obtained from synthesizing training plans from elite athletes can be readily applied in writing training plans for similar athletes. The concept of results-based practice is also applicable for sub-elite athletes, by quantitatively analysing the training plans of marathon runners who have achieved certain target times, such as a sub-4-h marathon. However, such an analysis has not yet been conducted.

This review aims to systematically and quantitatively analyse the last 12 weeks before a marathon as recommended in 92 sub-elite training plans. Whilst there are obvious limitations such as the subjective nature of such an analysis and no data on training plan effectiveness, we argue that the resultant information is useful for writing sub-elite marathon training plans and for testing hypotheses related to best practice training for millions of recreational runners.

## Methods

### Search Strategy

We obtained the training plans for this analysis from non-peer-reviewed sources, using the search term “marathon training”. Each plan that was considered had to incorporate a detailed week-by-week training schedule with the goal to complete a marathon race at the end of the program. Two researchers conducted this search, gathering the top 10 Google search results that were consistently found in both searches and that contained marathon training plans, which included sponsored plans by the world marathon majors, the sporting goods industry, and top running magazines. The same method was applied to the combined top 10 book results, again focusing on those that contained marathon training plans, from both the Amazon United States of America and United Kingdom stores at the time of searching (August 2022) [9, 10]. This process yielded 10 main online sources and 10 main print sources, some of which were accessible online. Among these 20 main sources of marathon training plans, certain sources contained between 1 and 17 distinct training plans targeting various time goals (e.g., ranging from sub-3:00 to sub-5:00 h finishing plans), diverse starting levels (e.g., novice, beginner, intermediate, or advanced), varied focuses (e.g., speed, or endurance), and different time or distance commitments per week. We included all these variations in our analysis, which resulted in 92 sub-elite marathon training plans, presented in Table 1, that we obtained and reviewed for further analysis. We selected this approach to ensure the relevance of our analysis by simulating the search behaviour of the vast number of recreational runners seeking marathon training plans. Two researchers from our team independently conducted this search, and their results were consistent, however, it is essential to acknowledge that search results can be influenced by factors beyond our control, such as geographical location, individual search histories, and search engine algorithm updates. To address this inherent uncertainty, we employed a strategy of analysing a diverse portfolio of plans from various sources. Nonetheless, we recognize that our search strategy remains a limitation.

**Table 1:** Overview of included marathon training plans

Plan title [references]	Training plan unit	Volume classification	Distance unit of plan	Plan duration (Weeks)
adidas [39]	Both	Low	km	21
ASICS [40]	Time	Low	mi	16
Boston Marathon—Level 1 [41]	Distance	Low	mi	20
Boston Marathon—Level 2 [42]	Distance	Middle	mi	20
Boston Marathon—Level 3 [43]	Distance	Middle	mi	20
Boston Marathon—Level 4 [44]	Distance	Middle	mi	20
Daniels—Novice [45]	Both	Low	mi	18
Daniels—2Q—Up to 40 mi (64 km) per Week [46]	Both	Low	mi	18
Daniels—2Q—41–55 mi (66–89 km) per Week [47]	Both	Middle	mi	18
Daniels—2Q—56–70 mi (90–113 km) per Week [48]	Both	High	mi	18
Daniels—2Q—71–85 mi (114–137 km) per Week [49]	Both	High	mi	18
Daniels—2Q—86–100 mi (138–161 km) per Week [50]	Both	High	mi	18
Daniels—2Q—101–120 mi (163–193 km) per Week [51]	Both	High	mi	18
Daniels—2Q—120 mi + (193 + km) per Week [52]	Both	High	mi	18
Daniels—4-Week—40 mi (64 km) per Week [53]	Both	Low	mi	26
Daniels—4-Week Cycle—41–55 mi (66–89 km) per Week [54]	Both	Middle	mi	26
Daniels—4-Week Cycle—56–70 mi (90–113 km) per Week [55]	Both	High	mi	26
Daniels—4-Week Cycle—71–85 mi (114–137 km) per Week [56]	Both	High	mi	26
Daniels—4-Week Cycle—86–100 mi (138–161 km) per Week [57]	Both	High	mi	26
Daniels—4-Week Cycle—101–120 mi (163–193 km) per Week [58]	Both	High	mi	26
Daniels—4-Week Cycle—120 + mi (193 km) per Week [59]	Both	High	mi	26
Daniels—18-Week [60]	Both	High	km	18
Daniels—12-Week [61]	Both	High	mi	12
Furman Institute of Running [62]	Both	Low	mi	18
Fitzgerald 20-Week [63]	Both	Low	mi	20
Fitzgerald 80/20—Level 1 [64]	Both	Low	mi	18
Fitzgerald 80/20—Level 2 [65]	Both	Middle	mi	18
Fitzgerald 80/20—Level 3 [38]	Both	High	mi	18
Galloway for Runners and Walkers [66]	Both	Low	mi	30
Hansons—Beginner [67]	Distance	High	km	18
Hansons—Advanced [68]	Distance	High	km	18
Higdon—Advanced 1 [69]	Distance	High	km	18
Higdon—Advanced 2 [70]	Distance	Middle	km	18
Higdon—Boston Bound [71]	Distance	Middle	km	13
Higdon—Intermediate 1 [72]	Distance	Middle	km	18
Higdon—Intermediate 2 [73]	Distance	Middle	km	18
Higdon—Marathon 3 [74]	Distance	Low	km	24
Higdon—Novice 1 [75]	Distance	Low	km	18
Higdon—Novice 2 [76]	Distance	Low	km	18
Higdon—Novice Supreme [77]	Distance	Low	km	30
Higdon—Personal Best [78]	Distance	Middle	km	30
Kastor—Abbott World Marathon Majors [79]	Both	Middle	mi	16
Kastor—20 Week [80]	Distance	Middle	mi	20
Marathon Handbook—3 Hour [81]	Distance	Middle	km	20
Marathon Handbook—3 Month [82]	Distance	Low	km	12
Marathon Handbook—4 Hour [83]	Distance	Middle	km	20
Marathon Handbook—6 Month [84]	Distance	Low	km	24
Marathon Handbook—16 Week [85]	Distance	Middle	km	16
Marathon Handbook—20 Week Advanced [86]	Distance	Low	km	20
Marathon Handbook—20 Week Advanced 2 [87]	Distance	Middle	km	20
Marathon Handbook—20 Week [88]	Distance	Low	km	20
Marathon Handbook—Couch to Marathon [89]	Distance	Low	km	24
McMillan—3 Month [90]	Both	Low	mi	12
McMillan—Novice [91]	Time	Low	km	12
McMillan—Novice/Intermediate [34]	Both	Low	mi	12
McMillan—Intermediate—Combo Runner [92]	Both	Middle	mi	12
McMillan—Intermediate—Speedster [93]	Both	Middle	mi	12
McMillan—Intermediate—Endurance Monster [94]	Both	Middle	mi	12
McMillan—Intermediate/Advanced—Combo Runner [95]	Both	High	mi	12
McMillan—Intermediate/Advanced—Speedster [35]	Both	High	mi	12
McMillan—Intermediate/Advanced—Endurance Monster [96]	Both	High	mi	12
McMillan—Advanced—Combo Runner [97]	Both	High	mi	12
McMillan—Advanced—Speedster [98]	Both	High	mi	12
McMillan—Advanced—Endurance Monster [99]	Both	High	mi	12
Bank of America Chicago Marathon [100]	Both	Low	mi	18
Nike Run Club [101]	Both	Low	mi	18
Nolan—Beginner [33]	Distance	Low	mi	16
Nolan—Intermediate [102]	Distance	Middle	mi	16
Nolan—Advanced [103]	Distance	Middle	mi	16
Pfitzinger—18 Week—Up to 55 mi (88 km) per Week [104]	Distance	Middle	km	18
Pfitzinger—12 Week—Up to 55 mi (88 km) per Week [105]	Distance	Middle	km	12
Pfitzinger—18 Week—55–70 mi (88–113 km) per Week [106]	Distance	High	km	18
Pfitzinger—12 Week—55—70 mi (88—113 km) per Week [107]	Distance	High	km	12
Pfitzinger—18 Week—70–85 mi (113–137 km) per Week [108]	Distance	High	km	18
Pfitzinger—12 Week—70–85 mi (113–137 km) per Week [109]	Distance	High	km	12
Pfitzinger—18 Week—85 + mi (137 + km) per Week [110]	Distance	High	km	18
Pfitzinger—12 Week—85 + mi (137 + km) per Week [111]	Distance	High	km	12
Runner’s World—Advanced—Sub 3:30 [112]	Both	High	mi	16
Runner’s World—Intermediate—3:30–4:30 [113]	Distance	Middle	mi	16
Runner’s World—Beginner—First Marathon [114]	Both	Low	mi	16
Runner’s World—Ultimate—Sub 3:00 [115]	Distance	High	mi	16
Runner’s World—Ultimate—Sub 3:15 [116]	Distance	Middle	mi	16
Runner’s World—Ultimate—Sub 3:30 [117]	Distance	Middle	mi	16
Runner’s World—Ultimate—Sub 3:45 [118]	Distance	Middle	mi	16
Runner’s World—Ultimate—Sub 4:00 [119]	Distance	Middle	mi	16
Runner’s World—Ultimate—Sub 4:30 [120]	Distance	Low	mi	16
Runner’s World—Sub 5:00 [121]	Distance	Low	mi	16
TCS London Marathon—Beginner [122]	Both	Low	mi	16
TCS London Marathon—Improver [33]	Both	Middle	mi	16
TCS London Marathon—Advanced [36]	Both	Middle	mi	17
Women’s Health Magazine [123]	Distance	Middle	mi	22
Women’s Running [124]	Both	Middle	mi	24

Training plan unit refers to how the training plan is written, either with sessions written based on distance or on time

*mi* miles, *km* kilometres

### Coding of Training Plans

Initially, we transcribed all plans into standardized Excel worksheets with a weekly countdown to the race distributed into days (Monday–Sunday) with each session split into distances. In our transcription, we removed the marathon race itself from the analysis and labelled the last 12 weeks before the race as weeks 11–0. For plans with a Monday race day, such as the Boston Marathon, the week before the race is week zero. Time-based plans were converted to distance using the plan’s descriptions and pace calculator. For instance, a 90-min fartlek run session with 11 repetitions of 1-min fast running and 1-min jogging was converted to distance by considering the average goal marathon time of the plan, the fartlek run’s description as an easy long run with hard and easy running repetitions and using the corresponding pace calculator to calculate the expected distance. When plans included a range, we used the middle value; for example, we transcribed a 16- to 20-mile-long run as an 18-mile run. Finally, we converted all distance measures into kilometres.

After converting all training plans into this standard format, we classified each part of a training session into one of five exercise intensity training zones for performance based on the model described by both Jamnick et al. and Seiler, with adjustments made to match the training descriptions included in the examined training plans as presented in Table 2 [11, 12]*.* We opted for the five-intensity training zone model because it blends the physiological reference points of the conventional three-zone model with added practicality, resulting in greater sensitivity and specificity in tailoring training for each athlete [12]. Here, it should be noted that when a training exercise was prescribed to be completed uphill, the intensity zone classification of the training session was increased by one zone. For example, for an uphill workout at a 10 k pace, instead of being in zone 4 representing a level 10 k pace exercise, the classification would be zone 5. Two researchers independently rated and agreed upon the intensity zones for each session, and any discrepancies were resolved by a third researcher (Additional file 1).

**Table 2:** Description of five endurance training intensity zones

Endurance training zone	Heart rate (% of HR_max_)	Rating of perceived exertion (RPE)	Relative to Thresholds	Typical accumulated duration	Example training sessions
Zone 1: Slow Endurance	< 72	1–2 (very light)	< Aerobic	1–6 h	Jogging, Warm-Up, Recovery
Zone 2: Extensive Endurance	73–80	3–4 (light)	Aerobic < Anaerobic	1–3 h	Long Run
Zone 3: Intensive Endurance	81–86	5–6 (moderate)	Aerobic < Anaerobic	50–90 min	Brisk, Half-Marathon, Marathon Pace, Tempo Run
Zone 4: Threshold Training	87–92	7–8 (hard)	~ Anaerobic	30–60 min	10 k Pace, Intervals, Threshold
Zone 5: High Intensity Training	> 93	9–10 (very hard)	> Anaerobic	15–30 min	Speed, Sprints, Mile Pace, 5 k Pace, Fast

Slightly modified from five zone models presented by Jamnick et al. and Seiler to better fit the descriptions accompanying the examined training plans [14, 15]. RPE here uses Borg CR 1–10 scale [125]. Aerobic threshold represents the rise of lactate above baseline, the gas exchange threshold, or the first ventilatory threshold. Anaerobic threshold represents the acceleration of blood lactate accumulation, the respiratory compensation point and/or the maximal lactate steady state

*hr* hour; *min* minutes, *HR* heart rate, *RPE* rating of perceived exertion

Next, we grouped the training plans into low, medium, and high volume categories. Since there were discrepancies in how the different training plans were “self-classified” in terms of beginner, intermediate, and advanced, we reclassified all the training plans based on the weekly running volume in the examined peak week of each plan. Research has suggested that training volume is correlated with marathon race times, so we believed this to be a suitable categorization method given the available data [13–15]. The ‘low volume’ category included all training plans whose peak week distance was under 65 km, ‘middle volume’ included those between 65 and 90 km, and ‘high volume’ those over 90 km. These distances were selected to create groups of similar size. Once categorized, we summarized the collected data quantitatively to determine the recommended training for various marathon levels, considering variables such as distance per week, runs per week, distance per session, longest run, and peak week.

### Analysis of Training Plans

We focused on comparing the examined parameters of the coded training plans across the three volume categories (low, middle, and high). To make the plans comparable despite varying durations (ranging from 12 to 30 weeks), we analysed and compared the last 12 weeks leading up to the marathon race. We also conducted additional analyses on the peak week, defined as the highest volume week within the last 12 weeks of each plan. For specific variables, we also analysed the progression, which was calculated as the delta from one week to the next and averaged over the relevant duration of the examined training plans. A delta negative value here means the distance, is decreasing from 1 week to the next. Here, we focused on the weeks up to and included the peak week as the build-up phase, while regarding the weeks after peak week as the tapering phase of the plan.

In general, parameters of interest for this analysis were weekly running volume in km, weekly long run distance, longest run included in the whole training plan, number of run sessions per week, distance covered in each session, cross-training, strength-training or rest days, and the intensity distribution in terms of distance covered in each of the five intensity zones per week. Intensity distributions were also converted to weekly percentages and averaged to make them comparable across the different absolute distances covered.

### Statistical Analysis

We transcribed the training plans into a Microsoft Excel document and analysed these using RStudio [16, 17]. We conducted statistical analyses using the R packages ‘doBy’ (version 4.6.16), and ‘stats’ (version 4.0.0) with a significance level of *p* < 0.05 [16, 18]. We also performed an analysis of variance (ANOVA) test with Tukey post-hoc correction on relevant variables to compare the different classifications of marathon training plans [19].

## Results

### Training Plan Characteristics

We divided the 92 marathon training plans into 30 high volume (peak weekly volume more than 90 km), 33 medium volume (peak weekly volume 65–90 km), and 29 low volume (peak weekly volume less than 65 km), respectively. The high-volume plans had a median target time of 3:15 h:min for the marathon, with the minimum being 3:00 h:min, the maximum being 3:30 h:min, and only 2 out of 30 training plans indicating a target time. On the other hand, the middle volume plan had a median target time of 3:52 h:min for the marathon, with the minimum being 3:00 h:min, the maximum being 4:30 h:min, and 8 out of 33 plans indicating a target time. Finally, the low volume plan had a median target time of 4:30 h:min for the marathon, with the minimum being 4:00 h:min, the maximum being 5:00 h:min, and only 3 out of 29 plans indicating a target time. There was no significant difference [F(2,89) = 1.03, *p* = 0.361] in the duration of the plans in the different groups, the high-volume plans consisted of 17.2 ± 4.8 weeks, the middle volume of 17.8 ± 3.9 weeks, and the low volume plans of 18.9 ± 4.7 weeks.

### Analysis of the Last 12 Weeks Before Race

**Fig. 1:**
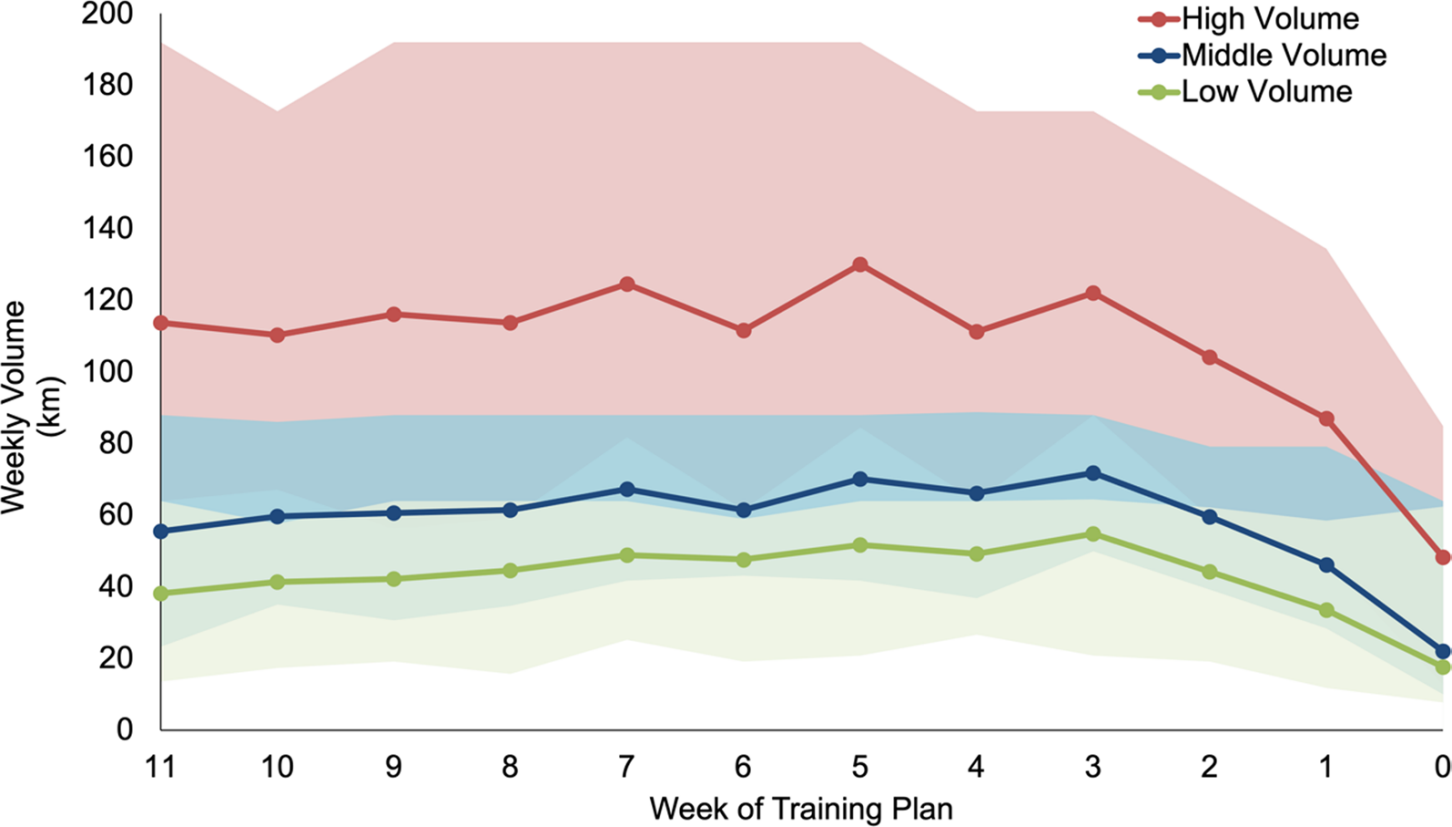
This line chart displays the weekly volume (in km) of the 12 weeks leading up to the race week, where week 0 refers to the week of the race and excludes the marathon race itself. The chart includes three ribbons indicating the different volume groups analysed: high, middle, and low. The lines in the chart represent the average value of the plans in each group, with the top and bottom of the bands indicating the maximum and minimum values within each group, respectively

**Fig. 2:**
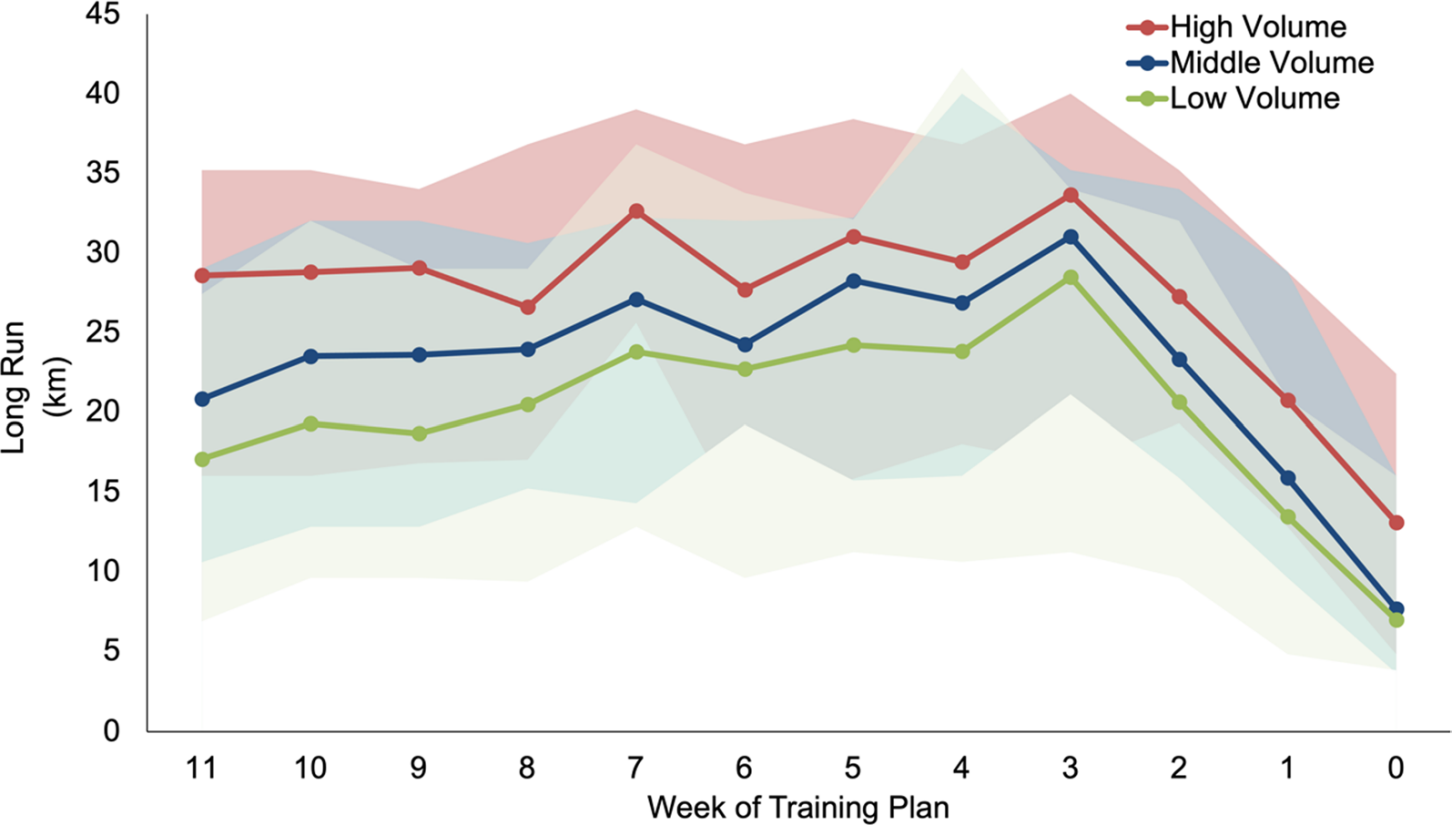
This line chart displays the weekly long run (in km) of the 12 weeks leading up to the race week, where week 0 refers to the week of the race and excludes the marathon race itself. The chart includes three ribbons indicating the different volume groups analysed: high, middle, and low. The lines in the chart represent the average value of the plans in each group, with the top and bottom of the bands indicating the maximum and minimum values within each group, respectively

Table 3 displays the average weekly distance, weekly long run, longest run, run sessions, cross training, strength training, rest days, and relative and absolute intensity distribution over the last 12 weeks before race day. The weekly volume (km) for the three different volume groups over the 12 weeks leading up to race week is displayed in Fig. 1, while Fig. 2 illustrates the weekly long run (km) in a comparable way.

**Table 3:** Average training characteristics of last 12 weeks of analysed training plans

Training variable	High volume	Middle volume	Low volume	ANOVA
Weekly Distance (km/week)	107.7 ± 38.4	58.5 ± 17.9	42.9 ± 14.1	F = 622.9
	*p*_Tukey_ = < .001 ‡§	*p*_Tukey_ = < .001 §		*p* = < .001 *
Weekly Long Run Session (km)	27.4 ± 7.1	23.0 ± 7.3	19.9 ± 7.5	F = 92.3
	*p*_Tukey_ = < .001 ‡§	*p*_Tukey_ = 0.001 §		*p* = < .001 *
Longest Run	35.2 ± 3.3	32.5 ± 3.8	30.9 ± 4.1	F = 15.2
(km)	*p*_Tukey_ = .002 ‡§			*p* = < .001 *
Run Sessions (runs/week)	6.8 ± 1.4	4.9 ± 0.9	4.1 ± 0.9	F = 599.9
	*p*_Tukey_ = < .001 ‡§	*p*_Tukey_ = < .001 §		*p* = < .001 *
Distance per Session (km/session)	16.5 ± 4.9	11.8 ± 3.0	10.6 ± 3.3	F = 246.8
	*p*_Tukey_ = < .001 ‡§	*p*_Tukey_ = < .001 §		*p* = < .001 *
Cross Training (sessions/week)	0.4 ± 0.7	0.6 ± 0.8	0.6 ± 0.9	F = 7.1
	*p*_Tukey_ = < .05 ‡§			*p* = < .001 *
Strength Training (sessions/week)	0.2 ± 0.7	0.3 ± 0.7	0.4 ± 0.7	F = 6.6
	*p*_Tukey_ = < .001 §			*p* = .001 *
Rest Day (days/week)	0.1 ± 0.3	1.3 ± 0.7	2.0 ± 0.9	F = 745.6
	*p*_Tukey_ = < .001 §	*p*_Tukey_ = < .001 §		*p* = < .001 *
Zone 1	14.3 ± 20.1	8.6 ± 12.8	4.5 ± 7.4	F = 40.8
(km/week)	*p*_Tukey_ = < .001 ‡§	*p*_Tukey_ = < .001 §		*p* = < .001 *
Zone 2	74.8 ± 38.9	35.7 ± 18.4	28.7 ± 16.7	F = 316.8
(km/week)	*p*_Tukey_ = < .001 ‡§	*p*_Tukey_ = < .001 §		*p* = < .001 *
Zone 3	10.4 ± 11.9	10.8 ± 9.7	7.4 ± 9.6	F = 11.3
(km/week)	*p*_Tukey_ = < .001 §	*p*_Tukey_ = < .001 §		*p* = < .001 *
Zone 4	5.1 ± 8.1	1.3 ± 3.3	1.2 ± 3.0	F = 63.9
(km/week)	*p*_Tukey_ = < .001 ‡§			*p* = < .001 *
Zone 5	3.2 ± 3.2	2.0 ± 2.3	1.0 ± 1.9	F = 66.5
(km/week)	*p*_Tukey_ = < .001 ‡§	*p*_Tukey_ = < .001 §		*p* = < .001 *
Zone 1	14.5 ± 20.5	13.7 ± 19.1	11.5 ± 18.4	F = 2.2
(% of km/week)				*p* = 0.11
Zone 2	67.5 ± 21.5	62.6 ± 26.7	66.7 ± 30.4	F = 3.7
(% of km/week)		*p*_Tukey_ = 0.03 §		*p* = 0.03 *
Zone 3	10.2 ± 11.3	18.1 ± 16.2	16.9 ± 22.1	F = 23.0
(% of km/week)	*p*_Tukey_ = < .001 ‡§			*p* = < .001 *
Zone 4	4.6 ± 7.0	2.1 ± 5.1	2.4 ± 6.3	F = 18.3
(% of km/week)	*p*_Tukey_ = < .001 ‡§			*p* = < .001 *
Zone 5	3.2 ± 3.4	3.4 ± 3.6	2.4 ± 4.9	F = 6.2
(% of km/week)	*p*_Tukey_ = 0.02 §	*p*_Tukey_ = 0.002 §		*p* = .002 *

Data presented as mean ± standard deviation. Zone classification based on descriptions found in Table 2

*ANOVA Significant difference (*p* < 0.05)

‡ANOVA Significantly different to middle volume (*p* < 0.05)

§ANOVA Significantly different to low volume (*p* < 0.05)

*km* kilometre, *ANOVA* analysis of variance

Here, high volume plans had higher weekly distances (107.7 ± 38.4 km), longer long runs (27.4 ± 7.1 km), more runs per week (6.8 ± 1.4 runs), and longer distance per session (16.5 ± 4.9 km) than middle and low volume plans. Predictably, low volume plans had shorter long runs (19.9 ± 7.5 km), fewer runs per week (4.1 ± 0.9 runs), shorter distances per session (10.6 ± 3.3 km), and more weekly rest days than high and middle volume plans (2.0 ± 0.9 days).

**Fig. 3:**
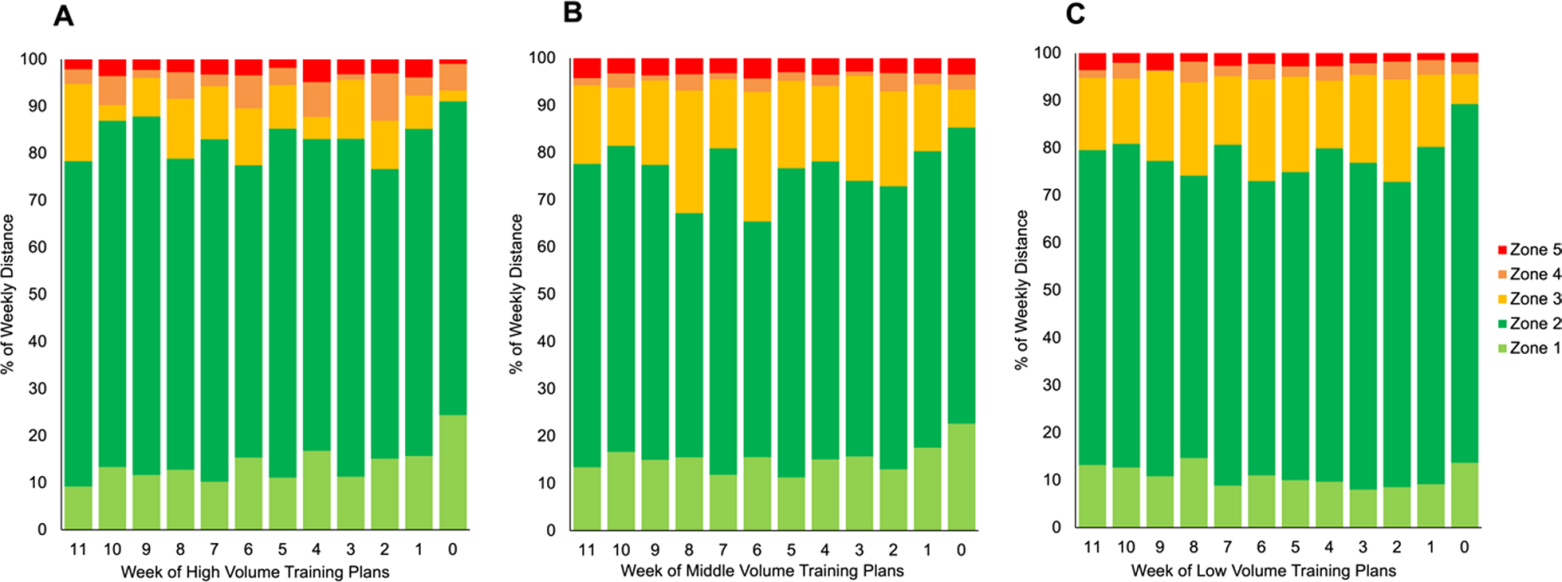
This bar chart displays the percentage of weekly volume distribution across the five intensity zones during the 12 weeks prior to the race week, where week 0 represents the week of the race with the race itself excluded. The chart is divided into three panels: **A** represents the high volume group, **B** represents the middle volume group, and **C** represents the low volume group. Refer to Table 2 for intensity zone descriptions

For the percentage of total weekly distance covered in each intensity zone, there were significant differences in all zones except zone 1. Surprisingly, the training plans varied within each group, indicating far less consensus than we might expect. High (67.5 ± 21.5%) and low (66.7 ± 30.4%) volume plans had significantly higher proportion of their weekly volume in zone 2 compared to the middle volume plans (62.6 ± 26.7%). For zone 3, the middle volume plans had the highest percentage (18.1 ± 16.2%), comparable to the low volume (16.9 ± 22.1%), while the high-volume group had significantly lower (10.2 ± 11.3%). The high-volume plans had significantly more of their weekly distance prescribed in zone 4 (4.6 ± 7.0%), compared to the middle (2.1 ± 5.1%) and low (2.4 ± 6.3%) volume plans. The low volume plans had the lowest percentage of their weekly volume in zone 5 (2.4 ± 4.9%) compared to the high (3.2 ± 3.4%) and middle (3.4 ± 3.6%) volume groups. These differences are presented in Table 3 and Fig. 3A–C.

Additionally, we conducted a detailed analysis of the intensity distribution for each individual training session, rather than solely focusing on the weekly volume. The average intensity distribution for these sessions aligns closely with the combined weekly intensity distribution mentioned earlier. On average, the training sessions exhibit intensity distributions of 17–67–8–4–4%, 17–60–17–2–4%, and 12–66–16–3–3% in zones 1–5 for the high, middle, and low volume groups, respectively.

### Analysis of Peak Week

Results for average weekly distance, weekly long run, longest run, number of run sessions, cross training, strength training, rest days, and the distribution of relative and absolute intensity during the week with the highest weekly volume (peak week) are presented in Table 4.

**Table 4:** Average training characteristics of peak week of analysed training

Training variable	High volume	Middle volume	Low volume	ANOVA
Peak week (week)	4.4 ± 1.4	4.0 ± 1.5	3.6 ± 1.4	F = 2.6
				*p* = 0.08
Weekly distance (km/week)	132.5 ± 34.5	75.5 ± 8.5	58.6 ± 6.4	F = 104.7
	*p*_Tukey_ = < .001 ‡§	*p*_Tukey_ = .005 §		*p* = < .001*
Weekly Long Run Session (km)	32.2 ± 6.2	31.2 ± 3.1	29.7 ± 4.9	F = 1.9
				*p* = 0.16
Cross Training (sessions/week)	0.4 ± 0.7	0.6 ± 0.7	0.6 ± 0.9	F = 0.8
				*p* = 0.46
Strength Training (sessions/week)	0.2 ± 0.6	0.3 ± 0.6	0.4 ± 0.7	F = 1.0
				*p* = 0.37
Rest Day (days/week)	0.0 ± 0.2	1.2 ± 0.7	1.9 ± 0.8	F = 68.6
	*p*_Tukey_ = < .001 ‡§	*p*_Tukey_ = < .001 §		*p* = < .001*
Run Sessions (runs/week)	7.1 ± 1.5	5.1 ± 0.8	4.2 ± 0.8	F = 55.8
	*p*_Tukey_ = < .001 ‡§	*p*_Tukey_ = .005 §		*p* = < .001*
Distance per Session (km/session)	20.2 ± 4.1	15.0 ± 1.6	14.3 ± 2.5	F = 35.9
	*p*_Tukey_ = < .001 ‡§			*p* = < .001*
Zone 1	13.9 ± 20.9	8.7 ± 12.1	5.3 ± 8.9	F = 2.5
(km/week)				*p* = 0.09
Zone 2	100.0 ± 40.3	51.2 ± 16.0	42.7 ± 19.1	F = 39.0
(km/week)	*p*_Tukey_ = < .001 ‡§			*p* = < .001*
Zone 3	11.9 ± 7.9	12.2 ± 7.2	8.1 ± 10.6	F = 2.1
(km/week)				*p* = 0.13
Zone 4	4.9 ± 9.0	1.0 ± 2.6	1.3 ± 3.0	F = 4.4
(km/week)	*p*_Tukey_ = 0.04 ‡§			*p* = 0.01 *
Zone 5	2.0 ± 2.6	2.5 ± 2.9	1.2 ± 2.0	F = 2.2
(km/week)				*p* = 0.11
Zone 1	10.9 ± 16.3	11.0 ± 15.3	9.4 ± 15.4	F = 0.1
(% of km/week)				*p* = 0.9
Zone 2	74.2 ± 18.6	67.9 ± 20.5	71.7 ± 30.4	F = 0.6
(% of km/week)				*p* = 0.57
Zone 3	9.9 ± 6.8	16.5 ± 10.4	14.7 ± 21.0	F = 1.9
(% of km/week)				*p* = 0.15
Zone 4	3.2 ± 5.6	1.3 ± 3.2	2.3 ± 5.2	F = 1.3
(% of km/week)				*p* = 0.26
Zone 5	1.7 ± 2.7	3.2 ± 3.6	2.0 ± 3.5	F = 1.8
(% of km/week)				*p* = 0.17

Data presented as mean ± standard deviation. Zone classification based on descriptions found in Table 2

*ANOVA Significant difference (*p* < 0.05)

‡ANOVA Significantly different to Middle Volume (*p* < 0.05)

§ANOVA Significantly different to Low Volume (*p* < 0.05)

*km* kilometre, *ANOVA* analysis of variance

Focusing on the peak training week of each running volume category, the high-volume training plans reached their peak at week 4.4 on average, while the middle volume group peaked at week 4.0, and the low volume group peaked even closer to race week at week 3.6. The high volume group had the highest weekly distance (132.5 ± 34.5 km), while the middle (75.5 ± 8.5 km) and low volume (58.6 ± 6.4 km) groups had lower weekly distances. Interestingly, the length of the long run session in peak week was similar for all three groups, at ~ 30–32 km showing that a long run of ~ 30 km in peak week is a common recommendation for all Marathon runners.

We also examined the breakdown of intensity zones for the peak week volume in each group and found there were no statistically significant differences among the three groups.

### Progression of Training Plan

To assess the progression changes of different marathon training plans, we analysed the weekly volume difference throughout the entire program. Our findings revealed that during the build-up phase leading up to the peak week, the high-volume plans prescribed an average weekly increase of 3 ± 1 km corresponding to an average of a 5 ± 3% increase from the week before in this phase, followed by a steep decrease of 21 ± 9 km per week or a 22 ± 12% reduction of weekly volume between peak week and race week during the tapering phase. In contrast, the middle volume plans increased by 3 ± 1 km or 7 ± 3% per week during build-up and decreased by 15 ± 6 km or 28 ± 12% per week during the tapering phase. Finally, for the low volume plans, during the build-up phase, the weekly increase was 2 ± 1 km or 9 ± 4%, while the volume decreased by 13 ± 6 km or 31 ± 13% during tapering. Surprisingly, this means that the high-volume plans had a gradual relative weekly change, while the low-volume plans showed more aggressive relative changes from 1 week to the next. Focusing specifically on the taper period following the peak week, all groups showed a particular stark decrease in the last week before the race with an average reduction of 46 ± 17% compared to the previous week for the high-volume plans, 54 ± 16% for the middle volume plans, and 50 ± 24% for low volume plans (Fig. 1).

## Discussion

In general, to achieve a target performance and to reduce the risk of detrimental effects of training, effective endurance running plans typically increase the frequency, duration, and intensity of training followed by a taper to maximize performance whilst reducing the possibility of adverse training effects [12]. There are many training plans recommendations that are used by probably millions of marathon runners, but we know little about how a typical marathon training plan recommendation for sub-elite athletes looks like and whether typical recommendations are consistent with current evidence from training intervention trials. The objective of this research was therefore to conduct a quantitative analysis of sub-elite marathon training plans, with a specific focus on the last 12 weeks before the marathon race, to provide a comprehensive overview of current sub-elite marathon training plan recommendations. While such an analysis has not been conducted before, other studies have sought to review the available literature for evidence-based research, study the training behaviour of recreational runners, or analyse elite training results-proven plans to make recommendations for marathon training [5, 6, 12, 20–24].

### How do the Recommended Recreational Training Plans Compare to Evidence-Based Research?

In 2007 Midgley et al. [5] concluded that there was little direct scientific evidence to identify the most effective training methods for enhancing long-distance running performance, with even less evidence specifically for the marathon distance. Since then, more work has been published to provide training guidelines for recreational runners and their coaches based on scientific evidence.

#### Running Training Methods

To improve performance in recreational runners, existing evidence recommends incorporating one to two high-intensity interval training sessions per week along with several sessions of moderate- and low-intensity continuous submaximal running into the training regimen [5, 20]. In the analysed plans, in the last 12 weeks before the marathon race, the high volume plans had an average of 7.8% of weekly volume in zone 4 and 5, while the middle volume plans had 5.5%, and the low volume plans had 4.8% at these intensities (Table 3). Despite the lack of clear understanding regarding the ideal volume and intensity of strength training for improving endurance running performance or preventing injury, it is advised to be included in a training regimen as well.

Another component of a training plan for which there is some empirical evidence is the taper before a race, or the intentional reduction in training volume before competition to improve running performance [25]. The varying tapering techniques used in research studies make it difficult to choose the best recommendation. According to a meta-analysis that investigated the impact of tapering on competitive athletes' performance, the most effective approach to maximize general performance gains is to implement a 2-week taper that involves an exponential reduction of training volume by 41–60%, without any changes to the intensity or frequency of training [26]. Intervention research focusing specifically on a 7-day taper found that the run taper group that reduced their training volume by 85% were 3% faster over a 5-km performance than the control group corresponding to an improved measured running economy [25]. With a focus specifically on the marathon distance, one study analysing the training activities of more than 158,000 recreational marathon runners determined that strict 3-week tapers are associated with better marathon performance compared to relaxed and shorter tapers [27]. In the analysed recreational training plans, peak week was found to be between 3 and 4 weeks out from race week, in line with a longer taper before the marathon race. Looking at the reduction in weekly volume following peak week until the marathon race, the tapers in the analysed plans are more gradual with a 22–31% weekly decrease. Focusing specifically on the last week before the race, the training volume decreases further by an average of 50% compared to the previous week in all plans. Among the three examined groups, the low volume training plans exhibit a shorter taper period, characterized by a peak week that occurs in closer proximity to the race week compared to the other groups.

#### Training Intensity Distribution

When designing a training plan, one crucial element is the distribution of training intensity across various intensity zones. Here a variety of different models are common including polarized, a pyramidal, and threshold models. Using a 3-zone intensity zone structure, a polarized training plan involves spending a significant percentage of time in zone 1 (75–80%) and in zone 3 (15–20%), with little or no time in zone 2, while a pyramid training plan has 70–80% of the volume in zone 1, with the remaining 20–30% in zone 2 and 3. Finally, when training follows the threshold model, the main focus, and therefore a higher proportion of overall volume, is on zone 2 training [20, 28]. Of these, polarized and pyramid training intensity distributions, that share a similar distribution of around 80% in low-intensity training but differ in how the remaining 20% is distributed, are the most recommended models. However, the evidence is inconclusive as to how best to optimize training [20, 28–30]. Based on these definitions and making it comparable, the last 12 weeks before the marathon of the analysed plans presented in Table 3 consist of a pyramid plan with high, middle, and low volume groups having 82–10–8%, 76–18–6%, and 78–17–5% in zone 1 and 2, zone 3, and zone 4 and 5, respectively. Previous intervention research has indicated that polarized training, with a distribution of 68–6–26% at low-lactate threshold-high intensity respectively, leads to the most significant improvements in various key endurance performance variables for well-trained endurance athletes compared to threshold, high intensity, or high volume training over a 9-week training program [30]. Conversely, a systematic review, which includes both intervention and observational studies, has found that highly trained distance runners tend to follow a pyramidal training intensity distribution approach, which is also related to high levels of performance and significant development of physiological determinants [28]. Another systematic review has analysed pyramidal training, polarized training, and threshold training and concluded that current evidence suggests pyramidal and polarized training to be more effective than threshold training, however among these no single optimal training intensity distribution has been established [29]. Although the inconclusive scientific evidence makes it challenging to recommend only one of these two models, recent research has explored the possibility of periodizing intensity distributions based on the stage of a runner's training cycle. For example, a 16-week pyramidal training plan followed by a 16-week polarized training plan results in the greatest improvement in performance, indicating that this could be a viable method to integrate differences in stimuli from both distributions [31].

### How Does the Training Behaviour of Recreational Runners Differ from the Recommended Training Plans?

To compare how established training recommendations align with the actual training behaviour of marathon runners, additional studies that describe these behaviours were considered. Gordon et al. examined the training characteristics of 97 recreational marathon runners including both males and females sub-grouped by different finishing times (2.5–3 h, 3–3.5 h, 3.5–4 h, 4–4.5 h, and > 4.5 h). This study found race speed for a marathon to be correlated with distance covered per training session, and weekly training distance [21]. Comparing these running behaviours, such as distance per week, distance per session, and the longest run of the plan, to the recommendations in the last 12 weeks before the marathon of the analysed plans, the training patterns of the 4–4.5 h group (56.2 km/week) was similar to the middle volume (58.5 km/week), and the > 4.5 h group (43.8 km/week) to the low volume plans (42.9 km/week). Only the weekly distance in the high volume plans of 107.7 km/week differed from the fastest finishing group of 2.5–3 h, which on average ran 91.7 km/week. When training for a marathon, it appears the actual training behaviours of recreational runners correspond well with the recommended most popular training plans for marathon performance [21].

For further analysis, Doherty et al. [22] performed a systematic review, meta-regression, and meta-analysis on 127 cohorts of runners to determine the relationship between training behaviours and marathon race performance. This analysis examined the average weekly running distance, number of weekly runs, maximum weekly running distance, number of runs ≥ 32 km in the pre-marathon training block, average running pace in training, longest run completed, and hours of running per week and found that increases in any one of these training parameters coincided with significant faster marathon finish times [22]. Based on the formulas they created, the marathon finish time calculated from the training recommendations for high volume training plans is 3:04, followed by 3:36 for the middle volume, and 3:50 for the low volume group. These predicted finishing times are faster than those suggested with the plans themselves and those predicted based on training behaviour [22].

### How do Training Plans for Recreational Runners and Elite Runners Differ?

To relate the examined recreational training plans analysed in this report to elite populations we compared our findings to the training habits of elite marathon runners. Billat and colleagues examined the training characteristics of top-class and high-level elite marathoners and while the absolute distances of these runners are very different from the plans investigated here, the average intensity distribution revealed 78% of the total weekly distance was run at velocities less than marathon pace, 5% at marathon pace, and 17% greater than their marathon pace, matching a typical polarized training model [23]. While the exact comparison cannot be made due to discrepancies between intensity distribution methods, considering the last 12 weeks before the marathon, the high volume group comes the closest to such a polarized model with an average of 82% of training at less than marathon pace (zone 1 and 2), and 8% greater than marathon pace (zone 4 and 5) while the middle and low volume groups follow a typical pyramid training model.

Additionally, giving further insights into the training behaviour of elite long-distance runners, Haugen and his colleagues published a review integrating scientific literature and results-proven practice to understand the training and development of elite long-distance runners [6]. For marathon runners, this review found the weekly running distance in the mid-preparation period to be between 160 and 220 km per week, again significantly higher than the examined training plans. The intensity distribution of this distance, in line with the last 12 weeks of our examined plans, was made up of ≥ 80% of the total running volume being performed at low intensity (zone 1 and 2), 5–15% at middle intensity (zone 3), and 5–15% at high intensity (zone 4 and 5) inversely related to the middle intensity training [6]. The tapering for these athletes started 7–10 days out from the main competition, whereas for our analysed plans the peak week was around 4 weeks out from the competition, with an additional pronounced decline the last week before the race (Table 4 and Fig. 1) [6].

Finally, research from Karp found that among analysed qualifiers for the United States of America Olympic marathon trials, the large majority of the training was performed at low intensity, with men running 74.8% and women running 68.4% of their weekly distance, at a pace slower than marathon race pace [24]. In more detail, the distribution of training intensity for men and women was 75–10–10–5–3% and 68–13–12–7–5% for intensities below marathon race pace and at marathon race pace, lactate-threshold pace, ≥ 10 k race pace, and ≥ 5 k race pace, respectively [24]. In comparison, the distribution of the last 12 weeks before the marathon data presented here is skewed towards the lower intensities for all volume classifications with 82–10–5–3% for high, 77–18–2–3% for middle, and 78–17–3–2% for low for intensities of zone 1 and 2, zone 3, zone 4, and zone 5, respectively.

### Limitations

Although our research has revealed new and potentially valuable insights that could assist coaches, athletes, and recreational runners in improving their training routines, there are several limitations to classifying the training plans in such a way that must be acknowledged. Firstly, it is important to recognize that unlike typical research databases such as PubMed, search outcomes from an Amazon or Google internet search may be impacted by variables outside of our influence, including location, personal search histories, and changes in search engine algorithms. To mitigate this inherent unpredictability, we focused on evaluating a diverse range of plans sourced from various places. Nevertheless, we acknowledge that our approach to searching still has its limitations. Secondly, the classification process involves subjective interpretation, as different training plans were written in various ways, making it necessary to analyse based on subjective decisions to ensure comparability. Moreover, the analyses here are limited to the last 12 weeks before the race, as certain training plans were only written for this duration. Additionally, both the subjective classification of the specific sessions into the five intensity zones and the classification of the training plan itself into low, middle, and high volume are subjective interpretations based on the range of training plans collected and the descriptions of the training sessions themselves.

Most training plans are not developed with a five-zone model in mind, and the intention of specific sessions may not always be apparent. Furthermore, we noticed discrepancies across the analysed training plans with different sources having varying definitions for commonly used phrases. We classified such sessions based on their descriptions in the plan rather than our understanding of the terms. For instance, several plans defined ‘steady’ runs differently, leading to varying categorizations. When steady was defined as a “purposeful pace … similar to marathon pace that helps to familiarize yourself to speeds you should set off on marathon day” [32], we classified this into zone 3, however for different plans steady runs were defined as the “runs to build the base for the rest of your training where conversations are still possible but only in shorter sentences” [33] or as a “continuous easy-medium pace” [34] which classified the sessions into zone 2. Some plans were also more detailed than others, and this may have affected the classification process. For example, one plan describes in detail a fartlek session starting with 20 min of easy running, then transitioning into 10 repetitions of 1 min hard where “you should be running fast enough that you cannot sustain the pace for more than a few minutes”, followed by 1 min at a very easy jog before completing the rest of the run at an easy running pace [35]; whereas another plan just includes 45 min of fartlek running with the explanation that “rather than running a set distance in a set time, you play with different running paces and distances until you feel you’ve completed the workout” [36]. Additionally, one plan might include 20 different types of sessions included in a plan, while another plan consisting entirely of easy and long runs [37].

Finally, as previously mentioned, another limitation results from converting time-based training sessions into distance-based measures, considering the variability of paces of runners that might intend to follow the plan which will in turn affect the distance covered in a given session. For example, as part of a tempo run, one source includes 30 min in zone 3 [38]. For an advanced goal marathon time of 3:00 h, based on the included pace descriptions, this would mean running this session at a recommended pace of 6:12 min per mile and therefore covering around 4.8 miles. However, for the same exercise, if the goal time is around 4:00 h, the pace for this tempo run would be around 8:10 min per mile meaning this session would cover 3.7 miles. While here for the analysis, we used the information available in the descriptions of the training plans to make the best calculation for how much distance would be covered in sessions written with only a time variable, there may still be considerable variability.

### Lessons Learned and Recommendations for Future Training Plans

The limitations identified in this analysis have highlighted significant differences in how training plans are developed and presented for recreational runners, which could potentially cause problems for those attempting to follow such plans. This lack of standardization in training plans makes it difficult to compare different plans, which limits the overall evidence base in this field. It is recommended that future training plans should be developed using consistent language and descriptions to ensure clarity and ease of understanding for those following the plans. By standardizing the language used to describe training sessions, runners can better understand what is expected of them during each session, and researchers can more effectively compare the effectiveness of different training plans. A clear and comprehensive training plan may incorporate the following elements: setting a target marathon time as the desired goal, utilizing a standardized 5-zone model for intensity recommendations, specifying the intended volume for training sessions, indicating the running speed in minutes per kilometre as the intensity measure, and providing information on the training plan structure, whether it is polarized, pyramidal, or follows a different framework. On top of that, this analysis has revealed limits in the existing evidence regarding the best tapering techniques and the optimal training intensity distribution for marathon performance with current research being inconclusive. Additionally, future training recommendations should consider how to optimize marathon preparation for different genders and age groups as well.

While our current study provides valuable insights into marathon training plans, we acknowledge that there are alternative approaches for analysis that could offer additional perspectives. One avenue for future research could involve a more detailed examination of training logs, as opposed to relying solely on pre-written training plans, utilizing a normalization process based on the percentage of the best world performance for a runner's age and gender. Such an approach not only permits a more nuanced evaluation of individual performances and training patterns, but also enables an assessment of effectiveness by correlating it with actual marathon performance outcomes.

## Conclusions

The training methods utilized by marathon runners based on best-practice and results-proven recommendations often advance faster than the science of training and performance. By examining and analysing a wide range of recommended plans for recreational runners and integrating best practices with a scientific approach, this research provides valuable insights into creating a marathon training plan. The five most important findings from this analysis include:

1. Typical weekly running volume in the last 12 weeks before a race averages to 108 km for high volume marathon training plans, 59 km for middle volume, and 43 km for low volume.
2. The analysed training plans, in the last 12 weeks before the race, have a pyramidal training intensity organization both in terms of weekly and session distance with 15–67–10–5–3%, 14–63–18–2–3%, and 12–67–17–2–2% of weekly in zones 1–5 distance for high, middle, and low volume respectively, incorporating both high intensity training sessions with continuous submaximal running into the training regimen.
3. By analysing the progression of the different plans during the build-up phase leading up to peak week, the high volume plans had the most gradual relative weekly increase of 5% corresponding here to 3.2 km, whereas the low volume plans showed a more aggressive progression, with a weekly increase of 9% corresponding here to 2.4 km.
4. Peak week analysis revealed that while the distances differed between the three groups, the intensity zone distribution was the same. Given the weekly long run session during peak week was consistent among all groups, there appears to be a consensus that the longest training run for a marathon should be 30–32 km independent of the distance you run per week.
5. All analysed training plans start with a gradual taper 3–4 weeks out from race week with a 22–31% weekly reduction between peak week and race week, with a further 50% reduction in the last week before the race compared to the previous week.

These findings could benefit researchers, athletes, and coaches by providing information on the types and extent of training that is recommended to recreational runners for a marathon. The review applies a unique approach to analysing training recommendations and highlights the distinct features of training methods, volume, and intensity, emphasizing the differences between groups of marathon runners. Although this method has apparent drawbacks, such as the subjective nature of analysing such recommendations, the inconsistency in plan duration, and the inability to measure the effectiveness of such training plans with marathon performance outcomes, it presents a viable solution to the lack of evidence-based training practices being used now. In general, this review provides fresh perspectives on aspects of marathon training that have received limited attention in scientific research and provides beneficial guidance for devising training programs tailored to runners of varying performance levels.

### Supplementary Information

**Additional file 1.** This file contains the the coded weekly data used for the analysis of the different marathon training plans including the weekly volume, weekly duration, weekly long run, the intensity distribution both in distance and in time, and the number of rest days planned for each week.

## Data Availability

All data generated from this research are available as electronic supplementary material that can be found in the online version at 10.1186/s40798-024-00717-5.

## Abbreviations

km: Kilometre
hr: Hour
min: Minute
ANOVA: Analysis of variance
F: F-statistic
*p*: *p*-Value
10 k: 10 Kilometre race
5 k: 5 Kilometre race
HR: Heart rate
RPE: Rating of perceived exertion
